# Rights That

**DOI:** 10.1093/ojls/gqae021

**Published:** 2024-07-04

**Authors:** Eleanor Eldridge

**Keywords:** rights, legal philosophy, private law, contract law, equity, tort

## Abstract

Hohfeld was acutely aware of the ‘potent tendency [of words] to control thought’. He was perhaps less aware of the power of syntax to do the same. Hohfeld’s tendency to express the content of duties and liberties in the syntactically restrictive *to ϕ* form has allowed a corrupted version of his analytical scheme to take root. That version takes as axiomatic that the content of any duty or liberty is the action or inaction of the duty bearer or liberty holder. Yet duties and liberties can (and do) pertain to matters other than the action or inaction of the duty bearer or liberty holder. This insight has a range of doctrinal implications.

## 1. Introduction

Wesley Newcomb Hohfeld famously cautioned that ‘chameleon-hued words are a peril both to clear thought and to lucid expression’.^1^ Similarly perilous, however, is chameleon-hued syntax. This article argues that Hohfeld’s uncharacteristically loose approach to the syntactic expression of duties and liberties has led to a serious and basic mistake about the fundamental nature of both. The mistake is the view that the content of all duties and liberties can be expressed in the form *to ϕ* (or *not to ϕ*), such that the basic content of any duty or liberty is the action or inaction of the duty bearer or liberty holder. This mistake, which is not native to his work, compromises Hohfeld’s signal contribution to analytical jurisprudence by rendering his scheme of jural correlatives non-exhaustive. By correcting the mistake, we preserve the integrity of Hohfeld’s scheme and realise an improved understanding of his first-order legal relations.

This article is structured in two parts. In the first part, the subject-action view is identified and exposed as a mistake. The subject-action view is the view that the basic content of any duty or liberty is the action or inaction of the duty bearer or liberty holder. It is first shown that whereas Hohfeld was a veritable pedant about diction, he did not bring that same obsessive disposition to syntax. Instead, Hohfeld used a variety of syntactic forms to express his claim-right/duty and liberty/no-claim-right relations. Second, it is explained that one of those syntactic forms (namely, the *to ϕ* or *not to ϕ* form) is restrictive. Grammatically, this is because the infinitive here cannot accommodate an overt subject. Legally, this means that the *to ϕ* form dictates that the content of any duty or liberty *must be* the action or inaction of the duty bearer or liberty holder. Third, it is shown that the view compelled syntactically by the indiscriminate use of the infinitive form has indeed taken root. It is now widely taken as axiomatic that the content of any Hohfeldian duty must be the action or inaction of the duty bearer (and that the content of any Hohfeldian liberty must be the action or inaction of the liberty holder).

Lastly in this part, the subject-action view is shown to be wrong. Hohfeld’s scheme was devised to admit any and all possible legal relations. It was not intended to restrict the scope of possible legal relations or to exclude some legal relations from its ambit. Yet people can (and do) assume legal duties in relation to the actions or inactions (or states) of third parties, non-persons and even—most radically—claim-right holders. For instance, Y can assume a duty to X that *Z* will do something (say, tend X’s flowerbeds). This is entirely different from a duty that Y might assume to X, say, to take steps to ensure that Z tends X’s flowerbeds. In both cases, Y is the duty bearer. But the content of the latter duty is Y’s action, whereas the content of the former duty is Z’s action. The same analysis applies *mutatis mutandis* to liberties. Claim-right/duty and liberty/no-claim-right relations are simply axes of, respectively, entitlement and non-entitlement; there are no analytical constraints on the content of these relations. This is the entitlement model.

In the second part, certain doctrinal implications of the entitlement model are explored. The entitlement model permits a distinction to be drawn between ‘performance’ and ‘non-performance’ duties. This distinction improves upon our understanding of consideration, revealing a well-entrenched error in this area. The performance/non-performance distinction also reveals a discrete category of case where specific relief is necessarily unavailable. Moreover, once it is understood that not all duties pertain to the action or inaction of the duty bearer, it is easier to reconcile certain cases of strict liability in tort with a duty- or rights-based account. In this regard, the rule in *Rylands v Fletcher* is considered.

## 2. The Entitlement Model

### A. Hohfeld’s Syntax

Hohfeld abhorred a homonym. His seminal 1913 article, ‘Some Fundamental Legal Conceptions as Applied in Judicial Reasoning’ (FLC), was animated by concern over the ‘inadequacy and ambiguity of [legal] terms’ and the ‘corresponding paucity and confusion as regards actual legal conceptions’.^2^ Hohfeld noticed that there are rights and there are *rights*,^3^ and he was anxious to afford each its own name. He identified four right relations or ‘correlatives’,^4^ each comprising two unique conceptions. Hohfeld declared these eight conceptions ‘the lowest common denominators of the law’.^5^ He also coupled the eight conceptions into four dynamics of ‘opposites’.^6^

The table is familiar:^7^

**Table:** 

*Jural correlatives*	claim-right^8^	liberty^9^	power	immunity
	duty	no-claim-right	liability	disability
*Jural opposites*	claim-right	liberty	power	immunity
	no-claim-right	duty	disability	liability

Hohfeld was painstaking about diction. But he did not bring that same intellectual rigour to matters of syntax. He oscillated between different syntactic forms to express the content (the subject matter) of duties and liberties. In particular, he articulated the content of the claim-right/duty relation alternatively using the *that p* formulation and the now conventional *to ϕ* formulation: ‘if X has a [claim-]right against Y *that* he shall stay off the former’s land, the correlative (and equivalent) is that Y is under a duty toward X *to stay off*’.^10^ And in a passage exemplifying the liberty/no-claim-right relation, Hohfeld engaged no fewer than three discrete syntactic forms (*that p*, *to ϕ*, *of ϕ-ing*):

[W]hereas X has a right or claim *that* Y … should stay off the land, he himself has the privilege *of entering* on the land; or, in equivalent words, X does not have a duty *to stay off* … [I]f … X has contracted with Y *to go on* the former’s own land, it is obvious that X has, as regards Y, both the privilege *of entering* and the duty *of entering*.^11^

Likewise:

[I]f X, a landowner, has contracted with Y *that* the former will not alienate to Z … the former has no privilege *of doing* the necessary acts [to alienate to Z]; or conversely, he is under a duty to Y *not to do* what is necessary to exercise the power.^12^

FLC, and Hohfeld’s subsequent work, is flush with further examples.^13^

### B. The Limitation of the Infinitive Form

The problem with Hohfeld’s relaxed attitude towards syntax is not the variation in his usage *per se*; his syntax is entirely appropriate for the duties and liberties he exemplified. The problem is that one of the syntactic forms he adopted, and which has come to be treated as definitional, is not capable of expressing the content of all duties and liberties. The infinitive form (a duty or liberty *to ϕ* or *not to ϕ*) has a peculiar grammatical limitation that constrains the possible content of these relations. The basic point is that the infinitive here cannot take an overt subject but must instead take what is known as a logical subject. Take the sentence: Wesley is under a duty *to write*. The logical subject of the infinitive ‘to write’ is Wesley. It is Wesley who is to do the writing. In a sentence of this form, it is not possible for ‘to write’ to take as its subject any person other than Wesley. It is not possible using the *to ϕ* form to construct a sentence wherein Wesley is the duty bearer but the content of the duty is anything other than the action or inaction of Wesley.^14^ The *to ϕ* form does not admit of such a sentence. In other words, where a duty or liberty is expressed in the *to ϕ* form, the content of the duty or liberty *must be* an action or inaction of the duty bearer or liberty holder.

A caveat applies in the case of the passive infinitive: Wesley is under a duty *to be admired* by Eleanor. In that case, the content of Wesley’s duty is Eleanor’s action (her admiring). But duties and liberties are rarely, if ever, expressed in the passive. In any event, the passive infinitive still dictates Wesley’s inclusion in the subject matter of the duty: Wesley is the logical object (it is he who is to be admired). Strictly, then, it is not possible using the *to ϕ* form to construct a duty or liberty whose content is neither an action or inaction *of* (active) nor *upon* (passive) the duty bearer or liberty holder. To avoid complicating the analysis, ‘infinitive’ and ‘*to ϕ*’ are used in this article to refer to the active infinitive in particular.

This subject/object limitation does not attend the syntactic form *that p.* Take the sentence: Wesley is under a duty *that* his child attends school. The duty bearer is Wesley. But the content of Wesley’s duty is that *his child* attends school. The duty here is about the action of—not Wesley—but his child. It is vital to notice that this duty is different from, say, a duty that Wesley *take measures to ensure* that his child attends school. The content of that duty would be the action of Wesley (his taking measures). But the content of the duty here is the action of Wesley’s child (the child’s attending school). This point is explored in more detail below. The immediate point is that the syntactic form *that p* is more flexible than the *to ϕ* form. This is because the finite *attends* can accommodate an overt subject: here, Wesley’s child. Unlike the *to ϕ* form, the *that p* form does not dictate that the content of the duty or liberty must be the action or inaction of the duty bearer or liberty holder.^15^

In the next section, it is shown that—consistently with the constraints of the *to ϕ* form—it is now taken as axiomatic that the subject matter of any Hohfeldian claim-right/duty or liberty/no-claim-right relation is the action or inaction of the duty bearer or liberty holder.

### C. The Prevalence of the Subject-Action View

Just about any Hohfeldian will tell you that a claim-right/duty relation always pertains to the action or inaction of the duty bearer and a liberty/no-claim-right relation always pertains to the action or inaction of the liberty holder.^16^ This can be termed the ‘subject-action view’, being the view that the subject of any duty or liberty (the duty bearer or liberty holder) must be the subject of the action or inaction which the duty or liberty treats. The subject-action view boasts a long and distinguished pedigree. Every example Hohfeld gave in FLC of the claim-right/duty or liberty/no-claim-right relation pertained, respectively, to the action or inaction of the duty bearer or liberty holder. Yet Hohfeld did not at any point explicitly commit himself to the subject-action view. Indeed, Finnis—after expressing the subject-action view as axiomatic—complains: ‘All Hohfeld’s statements and examples take this for granted: would that he had somewhere said it explicitly!’^17^ Rather, the subject-action view is made explicit and formal in the work of those later engaging with Hohfeld’s scheme.

The subject-action view was imposed on Hohfeld’s scheme almost immediately. Just a few years after FLC was published, Corbin stipulates that the content of any duty or liberty is the ‘action or forbearance’ of the duty bearer or liberty holder.^18^ Radin takes the same view.^19^ Glanville Williams likewise regards it as obvious that:

No one ever has a [claim-]right to do something; he only has a [claim-]right that someone else shall do (or refrain from doing) something. In other words, every [claim-]right in the strict sense relates to the conduct of another, while a liberty and a power relate to the conduct of the holder of the liberty or power.^20^

Finnis, in his article exposing professorial fallacies about Hohfeld, similarly takes as read that ‘A Hohfeldian claim-right can never be to do or omit something: it always is a claim that somebody else do or omit something: A’s claim-right is always to B’s action or omission, never to A’s’.^21^ Halpin, too, takes the subject-action view as axiomatic. He specifically relies on the view to discriminate between claim-rights and liberties:

The fundamental difference lies in a claim-right always covering conduct undertaken by another party (the duty holder), whereas a liberty always covers conduct undertaken by the liberty holder (the other party having no-[claim-]right that the conduct should not be performed).^22^

Goldberg and Zipursky similarly assert that ‘To say that X has a [claim-]right against Y is to say that X is entitled to Y’s adjusting her conduct in relation to X’.^23^ Indeed, a freshly annotated version of FLC was recently published containing the following editor’s note:^24^ ‘In the Hohfeldian terminology, a “[claim-]right” … is a mere legal entitlement that some other legal actor engage (a “positive” [claim-]right) or refrain from engaging (a “negative” [claim-]right) in some action.’

The examples are legion.^25^ The subject-action view obtains in well-nigh every account of Hohfeld’s first-order jural relations. It might fairly be contended that, in many of these instances, the primary subject of analysis was the correlativity of Hohfeld’s conceptions; statements about the possible content of these conceptions were merely incidental. Insofar as that is the context of the error, it reveals something about its source: the subject-action view would appear to stem from elision of the *content* and the *form* of duties and liberties. Whereas duties and liberties always take the form of a relation of entitlement or non-entitlement between the two parties to the relation, the content of these relations—their subject matter—need not pertain to the parties thereto.

There are some exceptions in the literature to the subject-action view. Holmes, along with Salmond and Williams, recognises that a person can make a promise about a matter other than her own conduct.^26^ A further set of exceptions is found in certain attempts to formalise Hohfeld’s scheme.^27^ This is, perhaps, not surprising; the spareness of formal systems and the exercise of expressing the content of Hohfeld’s conceptions as propositions are powerful prophylactics against the subject-action view. More surprising are exceptions found in moral philosophy:^28^ Thomson recognises that duties and liberties take the form *that p*, and explores ‘word-giving’ as a source of moral claim-rights that propositions are true (*viz*, that states of affairs obtain).^29^ Kramer makes the same point, observing that an assurance from one person to another that some day will be ‘splendidly sunny’ can, in certain circumstances, generate a moral duty that this be so.^30^

These writers do not, however, explore the particular point in any or sufficient depth. Indeed, even these writers do not escape the influence of the subject-action view.^31^ Holmes’s view that all legal duties are contingent duties *to pay* damages is characteristic of the subject-action view.^32^ Salmond and Williams similarly reframe all promises about matters other than the duty bearer’s conduct as promises *to pay* damages should the matter warranted not obtain.^33^ Anderson suggests that the subject matter of a duty or liberty is a state of affairs which the duty bearer or liberty holder ‘brings about’ or ‘makes true’ relatively to the claim-right holder or no-claim-right holder.^34^ For this reason, Anderson appears to think that X’s duty to Y *that p* will be unfulfilled if *p* obtains but not on account of X’s doing.^35^ Cullison likewise paraphrases a duty *that p* as a duty ‘to see to it’ *that p*.^36^ Allen asserts that ‘Hohfeld, in effect, specified the term “[claim-]right” to refer to a three-term relationship between … the [claim-]right-holder, the other party, and an act of the other party’.^37^ Thomson describes a liberty ‘of letting’ *p* obtain and formulates a breach of duty as the duty bearer’s ‘letting it fail to be the case that p’.^38^ Thomson’s position that duties and liberties are constraints and non-constraints on the behaviour of duty bearers and liberty holders is also typical of the subject-action view.^39^ Kramer’s work, too, at points betrays the subject-action view.^40^ Suffice to say, the subject-action view is pervasive and the exceptions only limited. It is now widely treated as axiomatic that the content of any Hohfeldian duty or liberty is the action or inaction of the duty bearer or liberty holder.

Of course, the subject-action view is not peculiar to Hohfeldians but pervades the rights jurisprudence more generally. *Justinian* provides that the ‘essence’ of an obligation is that ‘it binds another person to give, do, or perform something for us’.^41^ Austin stipulates that ‘all rights are rights to forbearances or acts on the part of the persons who are bound’.^42^ Hart, too, claims that the substantive content of ‘X’s legal right’ is that ‘Under a rule or rules of the system some other person Y is, in the events which have happened, obliged to do or abstain from some action’.^43^ But the broader phenomenon of the subject-action view is beyond the scope of this article. As will be shown, the subject-action view excludes certain first-order legal relations. Because of this, it is anathema to Hohfeld’s project *in particular*. A distinct virtue of Hohfeld’s project is its theoretical neutrality;^44^ Hohfeld devised an analytical tool for examining any and all jural relations. The subject-action view, therefore, is peculiarly offensive in the Hohfeldian tradition because it undermines the very object of Hohfeld’s work. The priority of this article is to restore the ability of this singular framework to assimilate *all* rights and thereby to restore Hohfeld’s claim to analytical ecumenism.

### D. The Entitlement Model

Both analytically and doctrinally, the subject-action view is wrong. It is necessary first to notice that Hohfeld billed his conceptions as ‘the lowest generic conceptions to which *any and all* “legal quantities” may be reduced’.^45^ Hohfeld purported to classify, not to cull, the legal relations. He did not intend to deny any legal relation membership to his framework; rather, he intended to admit each and every one to its proper taxonomical place.^46^ This is relevant because, as is shown in this section, the subject-action view *does* exclude certain first-order legal relations. It follows that the view represents a gloss on Hohfeld’s scheme and should not be accepted as native to his work.

The subject-action view provides that the subject matter of any claim-right/duty relation is the action or inaction of the duty bearer and that the subject matter of any liberty/no-claim-right relation is the action or inaction of the liberty holder. But this is wrong. People can (and do) assume duties in relation to the actions or inactions (or states) of third parties, non-persons and even—most radically—claim-right holders. Indeed, the content of *most liberties* is something other than the action or inaction of the liberty holder.

It is a truism that a Hohfeldian claim-right is always about the duty bearer’s obligation. Likewise, a Hohfeldian liberty is always about the liberty holder’s non-obligation. But the view that it follows that a claim-right must pertain to the action or inaction of the duty bearer and a liberty must pertain to the action or inaction of the liberty holder entails slippage. The valid, unsound argument in each case is this:

Claim-right/duty argument

*Premise.* The constitutive fact of a claim-right is the duty bearer’s obligation to the claim-right holder.

*Premise.* The subject matter of any duty bearer’s obligation is the action or inaction of the duty bearer.

*Conclusion.* The subject matter of any claim-right/duty relation is the action or inaction of the duty bearer.

Liberty/no-claim-right argument

*Premise*. The constitutive fact of a liberty is the liberty holder’s non-obligation to the bearer of the no-claim-right.

*Premise*. The subject matter of any liberty holder’s non-obligation is the action or inaction of the liberty holder.

*Conclusion*. The subject matter of any liberty/no-claim-right relation is the action or inaction of the liberty holder.

The trouble is that the second premise in each case is false. It is convenient to scrutinise the claim-right/duty argument, but the analysis applies *mutatis mutandis* to the liberty/no-claim-right argument. We have seen that, grammatically, the *to ϕ* form dictates that the subject matter of a duty must be the action or inaction of the duty bearer. But this does not reflect the analytical or doctrinal reality. Let us consider some examples.

Suppose A has a claim-right against B that B publish A’s manuscript. It is conventional to express the content of B’s duty in the *to ϕ* form: *to publish A’s manuscript*. The content of B’s duty can also be expressed in the form: *that B publishes A’s manuscript*. In a case such as this (where *p* is the action or inaction of the duty bearer), the content of B’s duty can be expressed in either the *to ϕ* or *that p* form. Indeed, this is likely why the fallacy attending the indiscriminate use of the infinitive form has largely evaded notice: for the vast majority of duties, the distinction does not matter. The content of most duties is the action or inaction of the duty bearer. This makes good sense. If a person is assuming (or having foisted upon her) a legal obligation, its subject matter should generally be something over which she has control.

But there *are* duties where *p* is not the action or inaction of the duty bearer. Those duties cannot be expressed in the *to ϕ* form. Suppose C enters into a contract with D under which the latter promises (for consideration) that it will rain tomorrow.^47^ C thereby acquires a claim-right against D that it rains tomorrow. While D is the person under the obligation, there is no sense in which D’s duty is about D’s action or inaction. D’s duty is accommodated in the basic form *that p*. Here, *p* is: *it rains tomorrow*. Technically, D has conferred on C an entitlement *vis-à-vis* D to an action of the atmosphere. This duty of D’s cannot be accommodated in the *to ϕ* form.

It is necessary to distinguish this type of obligation from a contingent contractual obligation. Suppose F bets E £100 that it will rain tomorrow. In that case, the better view is that E and F have each assumed contingent obligations.^48^ F has assumed an obligation to pay E £100 in the event that it does not rain tomorrow, and E has assumed an obligation to pay F £100 in the event that it does rain. Critically, however, whether it rains or does not rain tomorrow will not place one or the other party in breach. Suppose it *does not* rain. E will have a non-contingent claim-right against F that the latter pay her £100. But F has not breached any duty owed to E. E’s remedy is in debt, not damages; F was never under a duty to E that it would rain. In other words, E never had a claim-right against F that it would rain. E merely had a contingent claim-right that, if it were not to rain, F would pay E.

The position is different in the C–D example. There, the content of D’s duty *is* that it rains tomorrow. If it does not rain, D *is* in breach of her duty to C. The vital point to notice is that the fact D might not have any control over whether it rains does not mean D cannot assume an obligation for the same. The subject matter of a duty need not be the action or inaction of the duty bearer. All that is analytically necessary to constitute a duty is that, *vis-à-vis* the duty bearer, the claim-right holder is entitled to some state of affairs.

In the C–D example, the content of D’s duty is a natural event: rain. The content of a duty can also be the action or inaction of a third party. So G can have an entitlement *vis-à-vis* H to the action or inaction of J.^49^ Take the case where H is a landscaping company and contracts with G to do work on G’s flowerbeds using contractor J. By the contract, H might promise G that H will take reasonable care to ensure that J does not damage G’s flowerbeds. In that case, the subject matter of G’s claim-right against H is H’s action (*viz*, that H takes reasonable care to ensure …). But it is *also* possible that the contract properly construed contains a promise by H to G simply *that J will not damage G’s flowerbeds*. In that second case, the subject matter of H’s duty is *J’s* inaction (*viz*, that J does not damage …).

It bears emphasising that the foregoing does not derogate from the fundamental principle that it is the duty bearer alone who is obligated in a claim-right/duty relation. That is the immutable analytical form of a claim-right/duty relation. Take the last-mentioned example of G’s claim-right against H that J does not damage G’s flowerbeds. Insofar as that claim-right is concerned, it is H alone who is under the obligation to G. If J does damage G’s flowerbeds, it is *H* (not J) who violates G’s claim-right against H. Certainly, J might be under her own duty to either or both G and H that she does not damage G’s flowerbeds. But those would be entirely separate legal relations wherein J was the duty bearer.

The C–D and G–H examples above might appear academic, but the law is replete with claim-right/duty relations that do not pertain to the action or inaction of the duty bearer. Take *Dick Bentley Productions (Ltd) v Harold Smith (Motors) Ltd*.^50^ Bentley purchased a car from the defendant dealership. Prior to the conclusion of the contract, Smith (the dealer) told Bentley that the car had done just 20,000 miles since it had been fitted with a replacement engine and gearbox. This was untrue. The car had done a great deal more than 20,000 miles since being refitted. Bentley had therefore overpaid by at least £400. The Court of Appeal held that Smith’s statement was a warranty.^51^ That is, Smith had promised Bentley—under contract—that the car had done just 20,000 miles. That warranty had been breached. Accordingly, the trial judge’s award to Bentley of £400 by way of damages for the breach was not disturbed. That measure reflected the position Bentley would have been in had the contract been performed (*viz*, had the car done just 20,000 miles).^52^

Notice the analytical form of the warranty here. The warranty took the form of Smith’s duty to Bentley *that the car had done just 20,000 miles*. That is a duty *that* something be the case (*viz*, that the car had done just 20,000 miles). Such a duty has nothing to do with the action or inaction of Smith. It cannot be expressed in terms of Smith’s duty *to do* or *not to do* something.

*McRae v Commonwealth Disposals Commission* supplies another example.^53^ That case involved a contract between the plaintiffs and the Commonwealth^54^ whereby the former purchased from the latter a tanker off ‘Jourmaund Reef’, which tanker (and reef) did not in fact exist. The High Court of Australia held that the contract was not void for common mistake.^55^ Dixon and Fullagar JJ explained that ‘The only proper construction of the contract [wa]s that it included a promise by the [Commonwealth] that there was a tanker in the position specified’.^56^ Because there was no such tanker, the Commonwealth was in breach.^57^

It was vital to the measure of damages that the Commonwealth was in breach of its duty that a tanker existed, rather than simply a duty of delivery. If the breach complained of was non-delivery, the plaintiffs could not show loss: they could not show that, on the delivery counterfactual, the tanker delivered would have been of any worth.^58^ But because the plaintiffs could complain of the Commonwealth’s breach of its duty that a tanker existed, they could make out a *prima facie* case for substantive damages on the reliance basis.^59^ They could argue they incurred expenditure on a salvage operation in reliance on the Commonwealth’s promise that a tanker existed at or near the specified location. Because the tanker did not exist (*viz*, because of the Commonwealth’s breach), this expenditure was necessarily wasted. The Commonwealth was unable to displace that *prima facie* case because it could not show that on the non-breach counterfactual the expenditure would have been wasted.^60^ That is, the Commonwealth could not establish that had a tanker been in existence it would have been worthless.

Duties of the G–H form are familiar in the context of guarantees. In *Moschi v Lep Air Services*,^61^ for instance, the debtor/principal was under a contractual obligation to pay the creditor/guarantee a total of £40,000 by way of seven weekly instalments. The principal defaulted immediately, and by the fourth week had paid some £10,060 of the £24,000 then owing. The creditor terminated the contract for fundamental breach and proceeded against the guarantor for the full £40,000 less the £10,060 already paid. The guarantor relevantly argued that when the principal’s contractual obligations were prospectively discharged by termination, the obligation of the guarantor in relation to the remaining three weeks’ payments was also discharged. If the guarantor had assumed a contingent obligation to pay the guarantee in the event that the principal failed to meet a payment obligation (an E–F duty), this might have been right: the principal could not fail to meet payment obligations that were discharged and never arose.^62^ The House of Lords held, however, that the guarantor had assumed a duty *that* the principal would make its contractual payments to the guarantee.^63^ That is a duty of the G–H form. Accordingly, the guarantor was liable in damages for ‘the loss suffered by the creditor due to the principal debtor having failed to do what the guarantor undertook that he would do’.^64^ That loss represented the loss of the bargain attendant on the principal’s fundamental breach, rendering the guarantor liable for the full £40,000 less the instalments already paid.

These types of duties also arise outside of the contractual context, indeed outside of the private law context.^65^ The Education and Children’s Services Act 2019 (South Australia), for example, contains the following provision:

(1) Subject to this Act, a child of compulsory school age must attend at the school in which they are enrolled on every day … that instruction is provided for the child at the school.
(2) If a child of compulsory school age fails to attend school as required by subsection (1), each person who is responsible for the child is guilty of an offence.
  Maximum penalty: $5 000
(3) [Subsection (2) does not apply in certain cases.]
(4) In proceedings for an offence against this section, it is a defence for the defendant to prove that they took such steps as were reasonably practicable to ensure that the child to whom the offence relates attended school as required by subsection (1).^66^

It is necessary to address the offence and defence separately. Subsections (1) and (2) impose a duty on a person responsible for a child (such as the child’s parent^67^) to the state. The content of the duty is *that* the child attends school. The content of this duty is the action of the child (*viz*, the child’s attending school). The content of the duty is *not*, by way of contrast, the action or inaction of the parent. In other words, the parent cannot satisfy the duty by, say, taking reasonable steps to ensure that the child attends school.

But what of the defence in subsection (4)? Does that subsection not show that the parent can satisfy the duty by taking reasonable steps to ensure the child attends school? No. Subsection (4) provides a *defence* in proceedings brought to punish the parent for having violated the state’s claim-right *that* the child attends school. The defence does not circumscribe the scope of the parent’s duty. If the child does not attend school, the parent has breached her duty. Strictly, the defence confers on the parent an *immunity* from the state’s *power* to seek a punitive remedy for the parent’s violation of the state’s claim-right.^68^ Here, then, we see a further duty whose content is something other than the action or inaction of the duty bearer. Another example might be the duty imposed in various jurisdictions on a person responsible for a dog that the dog not attack a person.^69^

Perhaps most challenging is the next point. It follows inexorably from the analysis above that it is analytically possible (if empirically improbable) for K to have a claim-right against L that *K* (the claim-right holder) do or not do something. Suppose L promises K by deed that *K* will use Hohfeld’s scheme in her academic work after reading FLC. Perhaps L makes this covenant with the desire that it should demonstrate to K L’s confidence that K will find Hohfeld useful and thereby entice K to read the article.

It might be intuitive to construe this as a bet (analogous to the E–F example). But this is not the proper construction. L has not, in a legal sense, wagered something on the contingency that K uses Hohfeld. Legally, L has *promised* K that *K* will do something (*viz*, use Hohfeld). Accordingly, the relationship between the parties here is analogous to the C–D and G–H examples. That is, L has not assumed a contingent obligation to pay *if* K does not use Hohfeld after reading FLC. Rather, L has assumed an absolute obligation that K *will* use Hohfeld after reading FLC. If K does not use Hohfeld after reading FLC, L would be liable for damages for breach of covenant (not in debt). Accordingly, we see that the subject matter of a claim-right/duty relation can also be the action or inaction of the claim-right holder.

We have seen that commentators expressly dismiss this possibility. Finnis, for instance, states: ‘A Hohfeldian claim-right can never be to do or omit something: it always is a claim that somebody else do or omit something: A’s claim-right is always to B’s action or inaction, never to A’s.’^70^ He continues, ‘the axiom can readily be verified by asking oneself what could (logically) be the content of B’s correlative duty if A had (*per impossibile*) a claim-right [that A do] (or omit [doing]) something’.^71^ The answer to Finnis’s interrogative is this: the content of B’s duty would be that A do or not do the relevant something. That duty is certainly *unintuitive*, but it is analytically valid.

The above analysis applies *mutatis mutandis* to liberties. The subject matter of a liberty/no-claim-right relation can, of course, be the action or inaction of the liberty holder, but it can also be the action or inaction (or state) of a non-person, a third party or even the no-claim-right bearer. But notice something special about liberties here: not only is it the case that liberties *need not* pertain to the action or inaction of the liberty holder, but—flying in the face of the orthodox account—the vast majority of liberties *do not* pertain to the actions or inactions of the liberty holder. If we were to catalogue our liberties, most would relate to the actions or inactions of others or non-persons. For instance, M will usually have a liberty against N that it rains tomorrow. In other words, M does not owe N a duty that it not rain tomorrow. Indeed, M will usually also have a liberty against N that it *not* rain tomorrow. That is, M also does not owe N a duty that it rains tomorrow. Moreover, M will not—in the ordinary course—owe a duty to N that any of *N, O or P* sing in tune, take their tea with milk, call their mothers, *anything*. It follows that the failings of the subject-action view are particularly stark in the case of liberties: the subject-action view fails to accommodate *most* liberties.

This analysis, admittedly, splits from the natural English usage of ‘liberty’ (and ‘privilege’^72^). We do not typically speak of a person having a liberty against another in relation to something other than the liberty holder’s action or inaction. In this sense, the usage here is prescriptive rather than descriptive. Liberty/no-claim-right simply denotes a relationship of non-entitlement over a state of affairs. If M has a liberty against N *that p*, N has no entitlement against M *that not p*. A conventional liberty might be M’s liberty against N that she (M) writes on topics that bore N. A less conventional, but no less legitimate, liberty might be M’s liberty against N that O reads M’s work. In both cases, the analytical form of M’s liberty is that N has no entitlement against M *that not p*. Because this broader analytical conception sits uncomfortably with conventional usage, it might be preferrable to dub this right a ‘no-duty-not’.^73^ That is, M is under no duty to N that O *not* read M’s work. But ‘liberty’ is well entrenched as the jural opposite of the Hohfeldian duty, and ‘no-duty-not’ is unwieldy. Provided it is borne in mind that the use here is legal (and not natural), ‘liberty’ and ‘privilege’ should together be serviceable to denote all conceptions correlative to a no-claim-right. Hohfeld, of course, expressly accepted that his scheme broke with natural English usage insofar as it prescribed that liabilities could be advantageous.^74^

Finally, we have seen that it was vital to Hohfeld’s project that his scheme should accommodate all legal relations. The fact, then, that the subject-action view is unable to accommodate all legal relations undermines its claim to be a true account of Hohfeld’s scheme. It is true that, as already mentioned, all of the examples Hohfeld used in his 1913 article to showcase the claim-right/duty and liberty/no-claim-right relations concerned the action or inaction of the duty bearer or liberty holder. But this should not be taken to detract from the argument made in this section. Hohfeld’s article was short, offering only a small sample of examples. But especial support for the view advanced here can be found in a subtle nod to the entitlement model in a passage in his 1917 successor article to FLC. In the context of advancing his multital-paucital distinction, Hohfeld made the following remarks:

[L]et us suppose that A is the owner of Blackacre and X is the owner of Whiteacre. It may be assumed further that, in consideration of $100 actually paid by A to B, the latter agrees with A never to enter on X’s land, Whiteacre; also that C and D, at the same time and for separate considerations, make respective similar agreements with A … B may commit a breach of his duty, without involving any breach of C’s duty by C or any breach of D’s duty by D. For, obviously, the content of each respective duty differs from each of the others. *To make it otherwise C and D would have to be under a duty or duties (along with B) that B should not enter on X’s land. Even if that were the case, there would be said to be three separate duties unless B, C, and D bound themselves so as to create a so-called joint obligation.*^75^

Blink and you miss it. But in that passage Hohfeld raised the now radical notion of C and D being under duties to A *that B should not enter X’s land*. How did Hohfeld appraise that idea? Ambiguously. He simply said ‘Even if that were the case’. His contemplation of a duty whose content is the action of a third party, though, certainly tends in favour of the view that Hohfeld would endorse as analytically orthodox the view presented in this article.

## 3. Some Doctrinal Implications

### A. Performance and Non-performance Duties

The entitlement model permits a distinction to be drawn between what might be termed ‘performance duties’ and ‘non-performance duties’. We have seen that all duties take the form *that p*. We have also seen that *p* most commonly is the duty bearer’s action or inaction. But we have also seen that *p* can be something other than the action or inaction of the duty bearer. A duty might pertain to the action or inaction (or state) of a third party or non-person. A distinction can therefore be drawn between two types of duties: first, duties whose content is the action or inaction of the duty bearer—*performance* duties; and second, duties whose content is something other than the action or inaction of the duty bearer—*non-performance* duties.

Conventionally, all duties are presupposed to be susceptible of performance. For example, where a contracting party meets her contractual obligation, she is invariably said to have ‘performed’ the obligation. If ‘performance’ is merely meant to denote the satisfaction of an obligation, then this is of little moment. But it is helpful to discriminate between a duty’s being *satisfied* (or *met*) and a duty’s being *performed*. On the distinction advanced here, whereas all duties are in principle susceptible of satisfaction, only performance duties are susceptible of performance.

Take the following two promises by deed:

1. B promises A that B will tend A’s flowerbeds.
2. B promises A that it will rain tomorrow.

Both of these obligations are liable to *satisfaction*. If B tends A’s flowerbeds, her obligation to A is satisfied. If it rains tomorrow, B’s obligation to A is satisfied. But it is only the first promise that is liable to satisfaction *by performance*. The second promise cannot be satisfied by performance by B, and so should be classed as a non-performance promise. Performance is simply the mode of satisfying a certain type of obligation (*viz*, a performance obligation). As will be seen, the performance/non-performance distinction improves upon our understanding of consideration and the availability of specific relief.

### B. Consideration

The mistaken assumption that all promises are performance promises has distorted the principles governing consideration. It is undoubtedly correct that, in order for a promisee to enforce a promise in contract, consideration must move from the promisee.^76^ But the subject-action view has seen this principle extrapolated unsoundly.

*Chitty on Contracts*, for instance, starts with the trite proposition that ‘a person can enforce a promise only if they themself provided consideration for it’ before making the following move:

Thus if A promises B to pay a sum of money to B if C will paint A’s house and C does so, B cannot enforce the promise (unless, of course, they procured, or expressly or impliedly undertook to procure, C to do the work).^77^

Certainly, if the putative contract here is taken to be unilateral, then B cannot enforce A’s promise. It is self-evident that where the putative consideration for a promise is performance simpliciter the promisee must *perform*. But the parenthetical text alternatively contemplates a bilateral contract. The presumption is that, for B to have furnished a valid *promise consideration* here, it is necessary that B promised *to procure* C to do the work. This seems to be an incident of the subject-action view; it seems to presuppose that the only contractual duty B could assume relates to B’s own action or inaction. Yet, as we have seen, it is entirely competent for B to assume a duty to A that C will paint A’s house.

In the language of this article, the analysis in *Chitty* (and also in *Treitel*^78^) conflates the performance and the promise. The consideration (*viz*, the promise in the case of a bilateral contract) must move from the promisee, but—given there is such a thing as a non-performance promise—it is not necessary that the promisee promised *to perform*. It follows that Pollock’s canonical statement of consideration, famously endorsed by Lord Dunedin in *Dunlop Pneumatic Tyre Co v Selfridge Co*,^79^ is unduly narrow. Pollock’s definition is this: ‘An act or forbearance of the one party, or the promise thereof, is the price for which the promise of the other is bought, and the promise thus given for value is enforceable.’^80^ On the view advanced here, the promise for which another promise is bought *need not* be a promise of some act or forbearance of the first promisor.

It has been said that ‘to determine whether a promise can constitute consideration it is necessary to consider whether its performance would have been so regarded’.^81^ Let us call this the unilateral test. That is, we test whether the promise designed to support a bilateral contract is good consideration by asking whether the performance of that promise would support a unilateral contract. This might tend against the argument made here (it being obvious that a non-performance promise could never support a unilateral contract). But the unilateral test is bad.

The unilateral test is said to explain why ‘a mere promise to accept a gift cannot be consideration for the promise to make it’.^82^ It is also said to explain why a promisor’s promise to pay part of her existing debt to the promisee is not good consideration for the promisee’s promise to accept it in full discharge of the same.^83^ But, as will be seen, those promises both fail as consideration not because they could not support a unilateral contract but because the *satisfaction* of them ‘could not be viewed in the eyes of the law as a benefit to the original promisor or a detriment to the original promisee’.^84^

The real question in every case is whether the promise if *satisfied* would confer a fresh benefit on the promisee or impose a fresh detriment on the promisor. If so, all else being equal, the promise should constitute good consideration. This is a different question from whether the promise if *performed* would confer a benefit or impose a detriment. As we have seen, axiomatically, a non-performance promise cannot be performed. Where a person makes a non-performance promise, the fact that it could not constitute consideration in the case of a unilateral contract should not preclude its being recognised as consideration in the case of a bilateral contract.

Take again the case where, for a money consideration, B promises A that C will paint A’s house. On this construction, B’s promise is a non-performance promise. To ask whether B’s promise if *performed* would confer a benefit on A is to lead us into error: B’s promise is not one susceptible of performance. If we press on with the badly framed question, we: (i) consider the position if the contract were unilateral; (ii) notice that C’s painting the house could not be good consideration (it not being performance by B); and (iii) conclude that B’s promise that C paint the house cannot be good consideration here either. This is the answer that *Chitty* and *Treitel* give.

But when the question is framed properly (*viz*, if B’s promise were *satisfied*, would a benefit/detriment be made out?) we see that B’s promise consideration is good. It is (presumably) of benefit to A that her house is painted by C. Importantly, when the question is framed in this way, we still arrive at the correct result in relation to the obvious cases that *Chitty* and *Treitel* mention: a promisee’s accepting a gift does not, without more, confer a benefit on the promisor nor a detriment on the promisee; likewise, excepting perhaps where the debt is time barred, the promisee incurs no *fresh* detriment and the promisor no *fresh* benefit by the satisfaction of the promisee’s promise to pay part of an existing debt.

### C. Specific Relief

The performance/non-performance distinction also promotes a more sophisticated understanding of the availability of specific relief. Specific relief (meaning injunctive relief or a decree of specific performance) entails mandating that a duty bearer comply specifically with her duty. But it emerges that only *performance* duties are susceptible of the imperative ‘comply’. Implicit in an order that a duty bearer comply specifically with her obligation is the notion that the obligation is one which is susceptible of performance by the duty bearer.

It is trite that ‘Equity will not specifically enforce what cannot be done’.^85^ The meaning of ‘what cannot be done’ is various, and several aspects are well developed. However, the idea of a non-performance promise as a discrete category of obligation which ‘cannot be *done*’ has thus far been largely overlooked.^86^ The performance/non-performance distinction therefore reveals a discrete and novel factor (or sub-factor) going to the availability of specific relief. It is convenient briefly to canvass some of the existing doctrine before explaining how non-performance promises might fit in.

First, it is well established that a (performance) promise to transfer a right is insusceptible of performance where the promisor no longer holds the right that is the subject of the transfer. In *Ferguson v Wilson*, the Court of Appeal held that an order for specific performance of a share option was not available where all the shares had already been allotted.^87^ Likewise, in *E Johnson & Co Ltd v NSR Ltd*, the Privy Council advised that the defendant vendors could not be ordered specifically to comply with their obligation to convey where the subject land had been compulsorily acquired from them.^88^ As Kindersley V-C observed in obiter in *Seawell v Webster*, in ‘the extreme case of a vendor burning a title-deed[] the Court could not make a decree that he should deliver it up, and be imprisoned if he does not’.^89^

Second, a (performance) promise has been held insusceptible of performance where its performance would be contrary to a supervening obligation. In this context, that which ‘cannot’ be done seems more directly to pertain to permissibility than ability. Such promises are probably better treated as engaging the discrete matter of ‘illegality’ rather than ‘impossibility’. But because the impossibility analysis appears in leading academic work,^90^ it serves briefly to mention this class of promise here.

*Seawell v Webster* is a case of this type where it seems to have been held that it was not possible (rather than merely unlawful) for the promisor to perform. The Websters had relevantly contracted with Seawell under a building contract to pull down a party wall and erect a new one. The adjoining owner, however, referred the matter to arbitration under the Metropolitan Building Act 1855. The arbitral award provided that it was not necessary to pull down the existing wall and that it should instead be patched and underpinned. Section 85(8) of the Act relevantly provided that such awards ‘shall be conclusive, and shall not be questioned in any Court’. The Websters commenced works consistently with the award, and Seawell brought proceedings against them seeking specific performance. The Websters successfully argued that it was not possible for them to perform the relevant promise. Refusing to issue specific relief, Kindersley V-C reasoned as follows:

Assuming the defendants to be entirely in the wrong, doing everything in contravention of the agreement, still, when the Court is asked to restrain them from acting under the award, it is impossible for me to do so, since the defendants have not the power of doing that which it is said they ought to do.^91^

Third, a (performance) promise to procure some act by a third party has been held insusceptible of performance where it is impossible for the third party to do the relevant act. In *Udall v Capri Lighting Ltd (in liq)*,^92^ Whiting (a solicitor) had undertaken ‘to procure’ a charge from each of Roe and Gowling (his clients) on their respective residences. Udall subsequently sought an order of specific performance of Whiting’s undertaking. Before those proceedings were commenced, Gowing was adjudicated bankrupt and his residence sold with no surplus. At or around the same time that the proceedings were commenced, Roe was hospitalised with a terminal illness; he had since died. In the light of those facts, Balcombe LJ refused to make an order for specific performance. He cited the ‘old and trite law that the “court will not make any order in vain”’.^93^ His Lordship further observed: ‘It is unthinkable that a court should put a man at risk of imprisonment by making an order which it knows, at the time of making the order, is impossible of performance.’^94^

Notice that the promise by Whiting in *Udall* was subtly but importantly different from a non-performance promise. A promise that the promisor will *procure* security from another is a performance promise. The fact that another’s cooperation might be necessary in order for the promisor to perform does not detract from the fact that the basic subject matter of the promise is the action of the promisor: here, the procurance of security from the client. The action of the third party here is a step removed from the subject promise. In order for the promise in *Udall* to be a non-performance promise, it would be necessary for Whiting to have promised simply that his clients would give security.

The distinction can be showcased this way. Take the actual promise in *Udall* first: a promise that *Whiting will procure* security from the client.^95^ If the clients were voluntarily to produce security without any procurement by Whiting, then—on a strict if technical view—Whiting would not have performed the promise. Rather, Whiting would likely have had the promise discharged by frustration.^96^ But if the promise had instead been that *the clients give* security, then the clients’ voluntary production of security would work to *satisfy* Whiting’s obligation.

This distinction is important for present purposes because a performance promise that nonetheless relates indirectly to something other than the action or inaction of the promisor is not *ipso facto* insusceptible of specific enforcement. The promise of Whiting in *Udall* was of this form. If the clients in *Udall* were alive and not bankrupt, an order that Whiting specifically perform his promise would in principle have been available. Indeed, the judge at first instance *had* ordered that Whiting comply with his undertaking (there not having been evidence of impossibility before him).^97^ And on appeal Balcombe LJ specifically disclaimed any intention to criticise the judge’s order: ‘I do not say that the judge was wrong in making the order he did, since at the time he had no evidence as to impossibility and in those circumstances he was justified in ordering performance[.]’^98^

Where, however, a promise is a non-performance promise, the promise is *never* susceptible to specific enforcement. So, if Whiting had simply promised that the clients would give the charges, then—even if the clients were alive and not bankrupt—the promise would not be liable to specific enforcement (it not being a promise susceptible of performance). The performance/non-performance distinction thereby provides a clear and principled basis on which to discriminate between these deceptively similar obligations.

### D. Strict Liability in Tort

The insight that a duty need not pertain to the action or inaction of the duty bearer also offers a new way of thinking about strict liability in tort. Tort theorists disagree about whether all tortious liability is predicated on a breach of duty by the defendant. For Stevens, for instance, the defendant’s breach is the ‘gist’ of all torts.^99^ For others, at least certain torts are not based on any breach by the defendant, being instead principles of loss allocation.^100^ The subject-action view has compromised the debate. In particular, it has caused theorists to cast the net too narrowly when looking for a duty in certain cases of strict liability in tort. This has led the no-duty camp too readily to conclude that certain torts are not predicated on breach. It has also led the duty camp to nominate duties that do violence to the positive law. When it is recognised that duties take the form *that p*, it is easier to understand these strict liability torts as predicated on the defendant’s breach.

The claim made here is modest. It is not suggested that the instant analysis supplies an answer to any of the challenging theoretical or normative questions that attend strict liability in tort. Instead, the argument is that the entitlement model gives new doctrinal credibility to a duty- or right-based account of tort law. By clearing this doctrinal obstacle, more space is created for the theoretical and normative questions to be explored in earnest elsewhere.

The rule in *Rylands v Fletcher* is often cited as a pre-eminent example of a no-duty tort. It is the form of tortious liability that Stevens seems closest to conceding might not be predicated on breach.^101^ For that reason, and given constraints of space, it is the example focused on here. It stands to reason, however, that other putative no-duty torts (such as those for which a person is made accountable by the doctrine of vicarious liability) may be susceptible of similar analysis.

Myriad commentators view the rule in *Rylands v Fletcher* as an example of liability without any breach of duty.^102^ The defendants in the eponymous case contracted out the work of constructing a reservoir on their land for their mill. The contractors retained were in principle competent but in fact negligent in carrying out the works. Shortly after the works were completed, the reservoir burst and water flooded the colliery. The defendants were held liable for the loss caused to the claimant by the flooding.

In the Court of Exchequer Chamber, Blackburn J identified the question as ‘whether the duty which the law casts upon him, under such circumstances, is an absolute duty to keep [the mischievous thing] in at his peril, or is … merely a duty to take all reasonable and prudent precautions’.^103^ He determined that:^104^

[T]he person who for his own purposes brings on his lands and collects and keeps there anything likely to do mischief if it escapes, must keep it in at his peril, and, if he does not do so, is primâ facie answerable for all the damage which is the natural consequence of its escape.

The House of Lords affirmed the decision and reasoning of Blackburn J.^105^

The view that the defendants’ liability in *Rylands v Fletcher* was not animated by any breach of duty is challenging to reconcile with the positive law. It meets head-on with the reasoning of Blackburn J, who specifically expressed the principle in terms of the scope of the defendant’s ‘duty’.^106^ Those of the no-duty view have to argue that Blackburn J did not mean what he said.^107^

The view that the rule imposes liability without a breach is also difficult to maintain in the light of the House of Lords’ insistence that the rule has a ‘close relationship’ with, or more recently is a ‘sub-species’ of, nuisance.^108^ That the rule was not considered to be remarkable by those deciding it must also tend against the view that it imposed liability without breach. Lord Cairns LC twice described the principles governing the case as ‘simple’.^109^ And Blackburn J is recorded saying in argument some years later: ‘I wasted much time in the preparation of the judgment in *Rylands v Fletcher* if I did not succeed in showing that the law held to govern it had been law for at least 300 years.’^110^

If the no-duty view is difficult to square with the positive law account of *Rylands v Fletcher*, why is it so widely accepted? The answer is that it has also proved difficult to defend any *duty*-based account of the case. This is, at least in part, because the subject-action view unduly narrows the parameters of the search. Emphasis is routinely placed on the fact that the House of Lords did not suggest the defendants had a duty not to create or keep the reservoir.^111^ This much is true. But the claim that it follows that the defendants’ liability must have arisen otherwise than by breach of duty is bad; it proceeds on the false premise of the subject-action view. The basic mistake is the presupposition that any duty must pertain to the action or inaction of the duty bearer. The mistake carries as follows: (i) any duty of the defendants must have pertained to an action (or inaction) of them; (ii) the only relevant action of the defendants was keeping the reservoir on their land; (iii) the House held that the defendants had no duty that they not keep the reservoir on their land; therefore (iv) the liability of the defendants must have arisen otherwise than by breach of duty.

The subject-action view has similarly hamstrung duty-based accounts. Keating, for instance, argues that in cases such as *Rylands v Fletcher* the wrong is the defendant’s ‘unjustified’ or ‘unreasonable’ ‘failure *to repair* the injury done’.^112^ For the reason given by Goldberg and Zipursky, this is unpersuasive: a claimant is not required to prove the defendant failed to compensate the claimant in order to obtain a remedy under the rule.^113^ Like the no-duty accounts, this account suffers from an unstated commitment to the view that any duty must pertain to the action or inaction of the duty bearer: a duty *to ϕ* or *not to ϕ*. If the duty is not ‘not *to cause* injury’—so Keating’s thinking seems to go—the next best bet is a duty ‘*to repair* the injury done’.

A doctrinally coherent, duty-based account of the rule in *Rylands v Fletcher* is, however, forthcoming on the entitlement model. The account is this: a person is under a tortious duty *that* others not be harmed by the escape of a mischievous thing voluntarily accumulated on land in the course of a ‘non-natural’ use of the land being made for the duty bearer’s purposes.

Ripstein comes close to this account. He analyses the duty as one of ‘non-harm’ ‘through dangerous or “unnatural” activities’.^114^ Yet Ripstein’s account is still undermined by the subject-action view. Ripstein identifies the rule in *Rylands v Fletcher* as a *fault*-based tort, arguing that ‘Keeping things that are “known to be mischievous” if they escape is a species of fault’.^115^ For Ripstein, these cases involve ‘conclusive presumptions of negligence when injuries ensue from [very risky] activities’.^116^ Ripstein’s conceiving as wrong, even contingently, a person’s keeping of a mischievous thing is difficult to reconcile with the basic doctrinal point that there is no duty that one not keep mischievous things.^117^ Ripstein’s emphasis on the defendant’s conduct also sits uneasily with the fact that liability is imposed on the person for *whose purposes* the mischievous thing was brought or kept on the land (not necessarily the person who brought or kept it). The impulse to identify something as wrongful or faulty in the defendant’s *conduct* seems partly to be a function of the subject-action view: if the defendant is under a duty, then at the heart of that duty must be some *action* of the defendant (here, the defendant’s *keeping* the mischievous thing). The entitlement model frees the analysis from these action-based strictures and accommodates a duty-based account which is faithful to the positive law.

## 4. Conclusion

Hohfeld was acutely aware of the ‘potent tendency [of words] to control thought’.^118^ But he was perhaps less aware of the power of syntax to do the same. His tendency to express the content of duties and liberties in the syntactically restrictive *to ϕ* form has allowed an unduly narrow version of his analytical scheme to take root. That version takes as axiomatic that the content of any duty or liberty is the action or inaction of the duty bearer or liberty holder. This view compromises Hohfeld’s project, detracting from his important contribution to analytical jurisprudence. The genius of Hohfeld’s scheme is its ability to accommodate all legal relations. People can (and do) have duties and liberties whose content is other than the action or inaction of the duty bearer or liberty holder. Claim-right/duty and liberty/no-claim-right relations are axes of, respectively, entitlement and non-entitlement; there are no analytical constraints on the content of these relations. The doctrinal implications of this insight are several. Four were considered here. First, the entitlement model permits a useful distinction to be drawn between performance and non-performance duties. Second, this distinction improves upon our understanding of consideration, revealing a common mistake in this area. Third, the distinction refines our understanding of the limits of the availability of specific relief. Last, the entitlement model offers a new way of thinking about strict liability in tort. In particular, it offers a doctrinally coherent, duty-based account of the rule in *Rylands v Fletcher*.

## Footnotes

1 Wesley Newcomb Hohfeld, ‘Some Fundamental Legal Conceptions as Applied in Judicial Reasoning’ (1913) 23 Yale LJ 16, 29 (footnote omitted).

2 ibid 29.

3 Hohfeld was, of course, not the first to notice that ‘right’ assumes a variety of meanings in the law: see eg Jeremy Bentham, *The Works of Jeremy Bentham* (John Bowring ed, 1843) vol 3, 181, 217–18; John Austin, *The Province of Jurisprudence Determined* (John Murray 1832) 26; John W Salmond, *Jurisprudence* (Stevens & Haynes 1902) ch X. For an overview of some of these earlier efforts to disambiguate ‘right’, see eg William Herbert Page, ‘Terminology and Classification in Fundamental Jural Relations’ (1921) 4 American Law School Review 616; Julius Stone, *Legal System and Lawyers’ Reasonings* (Stevens 1964) 140–3.

4 For analysis of Hohfeld’s notion of ‘correlative’ (and ‘opposite’), see eg Glanville Williams, ‘The Concept of a Legal Liberty’ (1956) 56 Colum L Rev 1129, 1135–45; Alan D Cullison, ‘Logical Analysis of Legal Doctrine: The Normative Structure of Positive Law’ (1968) 53 Iowa L Rev 1209, 1223–33; Jonathan Gorman, *Rights and Reason* (Routledge 2014) 90–6; Andrew Halpin, ‘Correlativity and Its Logic: Assymetry Not Equality in the Law’ (2019) 32 CJLJ 83, 84, 93–6.

5 Hohfeld, ‘Some Fundamental Legal Conceptions’ (n 1) 58.

6 See n 4.

7 Adapted from Hohfeld, ‘Some Fundamental Legal Conceptions’ (n 1) 30.

8 ‘Claim-right’ is widely accepted in the literature as a serviceable alternative to Hohfeld’s ‘right’ or ‘claim’: see eg Williams (n 4); John Finnis, ‘Some Professorial Fallacies about Rights’ (1972) 4 Adel L Rev 377. ‘Right’ and ‘claim’, used individually, each have the distinct disadvantage of having another common usage: ‘right’ as a global term for the spectrum of claim-rights, liberties, powers and immunities; ‘claim’ for the act of bringing proceedings.

9 Consistently with conventional usage, this article principally uses ‘liberty’ (rather than Hohfeld’s ‘privilege’) to denote the conception which is correlative to a no-claim-right: see eg David Frydrych, ‘Rights Correlativity’ in Shyamkrishna Balganesh, Ted M Sichelman and Henry E Smith (eds), *Wesley Hohfeld a Century Later* (CUP 2022) 112 fn 5. The term ‘privilege’ is here reserved to denote an *exceptional* absence of a duty: see eg Williams (n 4) 1131–2.

10 Hohfeld, ‘Some Fundamental Legal Conceptions’ (n 1) 32 (emphasis added).

11 ibid 32 (emphasis added).

12 ibid 52–3 (emphasis added).

13 See eg ibid 32–3, 35–6, 39; Wesley Newcomb Hohfeld, ‘Fundamental Legal Conceptions as Applied in Judicial Reasoning’ (1917) 26 Yale LJ 710, 768–9; Wesley Newcomb Hohfeld, ‘Faulty Analysis in Easement and Licence Cases’ (1917) 27 Yale LJ 66, 71.

14 Action or inaction here includes the obtaining or maintaining of certain states or characteristics: Wesley is under a duty *to be tall*.

15 Notice too that, unlike the passive infinitive, the *that p* form also does not dictate that the content of the duty or liberty must be an action or inaction *upon* the duty bearer or liberty holder in passive: Wesley is under a duty that school is attended by his child.

16 See eg Finnis (n 8) 380, and others canvassed below.

17 ibid 380.

18 Arthur L Corbin, ‘Legal Analysis and Terminology’ (1919) 29 Yale LJ 163, 167–8. Corbin’s foreword to the third printing of the edited collection of Hohfeld’s work also presents the subject-action view as axiomatic or internal to Hohfeld’s scheme: Arthur L Corbin, ‘Foreword’ in Walter Wheeler Cook (ed), *Fundamental Legal Conceptions as Applied in Judicial Reasoning* (3rd edn, Yale UP 1964) ix.

19 Max Radin, ‘A Restatement of Hohfeld’ (1938) 51 Harv L Rev 1141, 1147–8.

20 Williams (n 4) 1145.

21 Finnis (n 8) 380 (emphasis omitted).

22 Halpin, ‘Correlativity and Its Logic’ (n 4) 93–4. See also Andrew Halpin, ‘Hohfeld and Rules’ in Balganesh, Sichelman and Smith (n 9) 144.

23 John CP Goldberg and Benjamin C Zipursky, ‘Hohfeldian Analysis and the Separation of Rights and Powers’ in Balganesh, Sichelman and Smith (n 9) 369.

24 Ted M Sichelman, ‘Some Fundamental Legal Conceptions as Applied in Judicial Reasoning’ in Balganesh, Sichelman and Smith (n 9) 24. See also 24 fn 19.

25 See also eg Anthony Dickey, ‘A Fresh Approach to the Analysis of Legal Relations’ (1974) 20 McGill LJ 261, especially 272–3; Pierre Schlag, ‘How to Do Things with Hohfeld’ (2015) 78 Law & Contemporary Problems 185, 201; Ted M Sichelman, ‘Very Tight “Bundle of Sticks”: Hohfeld’s Complex Jural Relations’ in Balganesh, Sichelman and Smith (n 9) 351.

26 Oliver Wendell Holmes Jr, *The Common Law* (Little, Brown 1881) 298–300; John Salmond and James Williams, *Principles of the Law of Contracts* (2nd edn, Sweet & Maxwell 1945) 6–7, 44–7.

27 See Alan Ross Anderson, ‘Logic, Norms, and Roles’ (1962) 4 Ratio 36; Cullison (n 4) 1226 fn 34; Alan Ross Anderson, ‘The Logic of Hohfeldian Propositions’ (1971) 33 U Pitt L Rev 29; Layman E Allen, ‘Formalizing Hohfeldian Analysis to Clarify the Multiple Senses to Legal Right: A Powerful Lens for the Electronic Age’ (1974) 48 S Cal L Rev 428.

28 More surprising because several subjects of moral philosophy—such as the ‘ought implies can’ debate (at least insofar as ‘ought’ is taken to be a duty) and the relationship between duties and reasons for action—would seem to presuppose that duties take the form *to ϕ*.

29 Judith Jarvis Thomson, *The Realm of Rights* (Harvard UP 1990) 294–321.

30 Matthew H Kramer, *Moral Realism as a Moral Doctrine* (Wiley-Blackwell 2009) 75–6. See also Matthew H Kramer, ‘Moral Rights and the Limits of the Ought-Implies-Can Principle: Why Impeccable Precautions Are No Excuse’ (2005) 48 *Inquiry* 307, 352 en 8.

31 In this regard, notice that the use of the *that p* form is necessary but not sufficient to resist the subject-action view. Relatedly, Gorman uses the *that p* form but does not explicate the meaning of *p*: Gorman (n 4) chs 7, 11.

32 See OW Holmes Jr, ‘The Path of the Law’ (1897) 10 Harv L Rev 457, especially 462.

33 Salmond and Williams (n 26) 7, 45–7.

34 Also ‘does’ or ‘see[s] to’: Anderson, ‘Logic, Norms, and Roles’ (n 27) 42–6; Anderson, ‘The Logic of Hohfeldian Propositions’ (n 27) 31–2, 37–8.

35 Anderson, ‘The Logic of Hohfeldian Propositions’ (n 27) 33–4. But note that Anderson is here speaking about what he terms ‘Hohfeldian propositions’ (and not duties and liberties *per se*).

36 Cullison (n 4) 1232.

37 LE Allen (n 27) 433. He makes an analogous claim about liberties: 434.

38 See eg Thomson (n 29) 44, 111.

39 ibid especially 64–6, 77–8.

40 See Matthew H Kramer, ‘Rights Without Trimmings’ in Matthew H Kramer (ed), *A Debate Over Rights: Philosophical Enquiries* (OUP 2000) 9–10, 13, 22, 109. See also eg Matthew H Kramer, *Freedom of Expression as Self-Restraint* (OUP 2021) 10–11, cf the treatment of liberties 11–12; Matthew Kramer, ‘Shyamkrishan Balganesh, Ted Sichelman, and Henry Smith (eds), *Wesley Hohfeld a Century Later*’ (2023) 60 *Society* 639, 640.

41 Alan Watson (ed, tr), *The Digest of Justinian* (University of Pennsylvania Press 2009) vol 4, 155.

42 John Austin, *Lectures on Jurisprudence* (Robert Campbell ed, 4th rev edn, John Murray 1873) vol 1, 406 (emphasis omitted).

43 HLA Hart, ‘Definition and Theory in Jurisprudence’ in HLA Hart (ed), *Essays in Jurisprudence and Philosophy* (OUP 1983), 35. See also eg Albert Kocourek, ‘Reply to Paper of Professor Corbin’ (1921) 4 American Law School Review 614.

44 See eg Andrew Halpin, ‘The Value of Hohfeldian Neutrality when Theorising about Legal Rights’ in Mark McBride (ed), *New Essays on the Nature of Rights* (Hart Publishing 2017). See also Arthur L Corbin, ‘Jural Relations and their Classification’ (1921) 30 Yale LJ 226, 237–8; Kramer, ‘Rights Without Trimmings’ (n 40) especially 22; Schlag (n 25) 189–90.

45 Hohfeld, ‘Some Fundamental Legal Conceptions’ (n 1) 59 (emphasis added). See also eg Walter Wheeler Cook, ‘Hohfeld’s Contribution to the Science of Law’ (1919) 28 Yale LJ 721, 723–4.

46 See eg Kramer, ‘Rights Without Trimmings’ (n 40) 30.

47 Holmes gives this example: Holmes, *The Common Law* (n 26) 298–300.

48 Kramer conceptualises these as ‘quasi-liabilities’: see Matthew H Kramer, ‘Looking Back and Looking Ahead: Replies to the Contributors’ in Mark McBride and Visa AJ Kurki (eds), *Without Trimmings: The Legal, Moral, and Political Philosophy of Matthew Kramer* (OUP 2022) 367–8. See also Kramer, *Moral Realism as a Moral Doctrine* (n 30) 78–82.

49 Let us skip the letter ‘I’ to avoid unnecessary confusion.

50
*Dick Bentley Productions Ltd v Harold Smith (Motors) Ltd* [1965] 1 WLR 623 (CA).

51 ibid 627–9.

52
*Robinson v Harman* (1848) 1 Ex 850, 154 ER 363.

53
*McRae v Commonwealth Disposals Commission* (1951) 84 CLR 377 (HCA).

54 See ibid 419.

55 ibid 402–10.

56 ibid 410. See also 412.

57 ibid 410.

58 ibid 411–12.

59 ibid 412–14.

60 ibid 414.

61
*Moschi v Lep Air Services Ltd* [1973] AC 331 (HL).

62 See ibid 344–6 (Lord Reid), 350–1 (Lord Diplock).

63 ibid 345–6 (Lord Reid), 347–9, 351 (Lord Diplock), 352–3, 355–7 (Lord Simon), 359 (Lord Kilbrandon). For another example of a guarantee in this form, see *Sunbird Plaza Pty Ltd v Maloney* (1988) 166 CLR 245 (HCA).

64
*Moschi* (n 61) 345 (Lord Reid).

65 There is, of course, some debate as to whether Hohfeld’s scheme is applicable to public law. The better (and increasingly dominant) view is that it is: see eg Kit Barker, ‘Private Law, Analytical Philosophy and the Modern Value of Wesley Newcomb Hohfeld: A Centennial Appraisal’ (2018) 38 OJLS 585, especially 594–6, 599–600.

66 s 68.

67 Education and Children’s Services Act 2019 (SA), s 3(2).

68 It might be objected that the defence here really works to circumscribe the scope of the duty: relatedly, see eg Matthew H Kramer and Hillel Steiner, ‘Theories of Rights: Is There a Third Way?’ (2007) 27 OJLS 281, 292. On that view, subs (4) would operate to redefine the parent’s duty as one capable of satisfaction by *either* the child attending school consistently with subs (1) *or* the parent taking such steps as are reasonably practicable to ensure the child attends school consistently with subs (1). If that view be preferred, it is easy to contemplate the same legislative provision but without subs (4).

69 See eg Companion Animals Act 1998 (NSW), s 16(1); Domestic Animals Act 1998 (Vic), s 29; Domestic Animals Act 2000 (ACT), s 49A; cf perhaps Dangerous Dogs Act 1991, s 3(1).

70 Finnis (n 8) 380 (emphasis omitted).

71 ibid 380 (footnote omitted).

72 See n 9.

73 See relatedly eg Williams (n 4) 1135–9.

74 See Hohfeld, ‘Some Fundamental Legal Conceptions’ (n 1) 54 fn 90.

75 Hohfeld, ‘Fundamental Legal Conceptions as Applied in Judicial Reasoning’ (n 13) 742 (emphasis added).

76 See eg *Thomas v Thomas* (1842) 2 QB 851, 859; 114 ER 330, 333–4; *Tweddle v Atkinson* (1861) 1 B & S 393, 398, 99; 121 ER 762, 764.

77 Hugh G Beale (ed), *Chitty on Contracts* (35th edn, Sweet & Maxwell 2023) [6-040].

78 See Edwin Peel, *Treitel: The Law of Contract* (15th edn, Sweet & Maxwell 2020) [3-023].

79
*Dunlop Pneumatic Tyre Co Ltd* [1915] AC 847 (HL) 855.

80 Sir Frederick Pollock, *Principles of Contract* (8th edn, Stevens & Sons 1911) 175.

81
*Re Dale* [1994] Ch 31 (Ch), 38 (Morritt J). See also Beale (n 77) [6-009]; Peel (n 78) [3-008], the latter text expressing it only ‘as a general rule’.

82 Peel (n 78) [3-008]. See likewise Beale (n 77) [6-009].

83 Peel (n 78) [3-008]. See likewise Beale (n 77) [6-009].

84 See Beale (n 77) [6-009].

85 JD Heydon, MJ Leeming and PG Turner, *Meagher, Gummow and Lehane’s Equity: Doctrines and Remedies* (5th edn, LexisNexis Butterworths 2015) [20-140], citing *Ferguson v Wilson* (1866) LR 2 Ch App 77 (CA).

86 Aiden Lerch makes a similar argument in his doctoral research on injunctive relief in tort law. Lerch’s argument, however, is predicated on the view that these are ‘primary liability’ cases and do not involve an anterior duty.

87
*Ferguson v Wilson* (n 85) 86–7 (Turner LJ), 91 (Cairns LJ).

88
*E Johnson & Co (Barbados) Ltd v NSR Ltd* [1997] AC 400 (PC) 411 (Lord Jauncey).

89
*Seawell v Webster* (1859) 29 LJ Ch 71 (Ch) 73.

90 See Heydon, Leeming and Turner (n 85) [20-140]. *Snell’s Equity* includes *Warmington v Miller* [1973] QB 877 (CA) under both ‘illegality’ *and* ‘impossibility’: John McGhee and Steven Elliott (eds), *Snell’s Equity* (34th edn, Sweet & Maxwell 2020) [17-018], [17-046].

91
*Seawell v Webster* (n 89) 73.

92
*Udall v Capri Lighting Ltd* [1988] QB 907 (CA).

93 ibid 915, quoting *New Brunswick Co v Muggeridge* (1859) 4 Drew 686, 699; 62 ER 263, 268.

94
*Udall* (n 92) 915.

95 Conventionally, a promise *to procure*.

96 Although, properly construed, the actual promise in *Udall* was probably a promise that, in the absence of the clients voluntarily tendering security, Whiting would procure it. On that construction, Whiting’s promise was contingent (and the contingency would be foreclosed by the clients voluntary issuing security).

97
*Udall* (n 92) 912–14.

98 ibid 915.

99 See Robert Stevens, *Torts and Rights* (OUP 2007) 2.

100 See Peter Jaffey, ‘Duties and Liabilities in Private Law’ (2006) 12 Legal Theory 137; Peter Jaffey, ‘Liabilities in Private Law’ (2008) 14 Legal Theory 233, especially 240; John CP Goldberg and Benjamin C Zipursky, ‘The Strict Liability in Fault and the Fault in Strict Liability’ (2016) 85 Fordham L Rev 743, 757ff.

101 See Stevens (n 99) 111–13, 299. See relatedly James Goudkamp and John Murphy, ‘The Failure of Universal Theories of Tort Law’ (2015) 21 Legal Theory 47, 80–3.

102 See eg Carleton Kemp Allen, *Legal Duties and Other Essays in Jurisprudence* (Clarendon 1931) 193–4; John Murphy, ‘Rights, Reductionism and Tort Law’ (2008) 28 OJLS 393, 400; Jaffey, ‘Liabilities in Private Law’ (n 100) 240; Goldberg and Zipursky (n 100) 757–71; Stephen A Smith, *Rights, Wrongs, and Injustices: The Structure of Remedial Law* (OUP 2019) especially 258–60, 268. See also Donal Nolan and Andrew Robertson, ‘Rights and Private Law’ in Donal Nolan and Andrew Robertson (eds), *Rights and Private Law* (Hart Publishing 2012) 16–17.

103
*Fletcher v Rylands* (1866) LR 1 Ex 265 (Ex Ch) 279.

104 ibid 279.

105
*Rylands v Fletcher* (1868) LR 3 HL 330 (HL).

106
*Fletcher v Rylands* (n 103) 279.

107 Allen describes Blackburn J’s use of the word ‘duty’ as ‘unfortunate’: CK Allen (n 102) 193.

108
*Cambridge Water Co v Eastern Counties Leather plc* [1994] 2 AC 264 (HL) 297 (Lord Goff); *Transco plc v Stockport Metropolitan BC* [2003] UKHL 61, [2004] 2 AC 1 [9] (Lord Bingham).

109
*Rylands v Fletcher* (n 105) 388, 339.

110 See *Ross v Fedden* (1872) 26 LT 966 (QB) 968. See also *Cambridge Water* (n 108) 299 (Lord Goff).

111 See eg Goldberg and Zipursky (n 100) 761–2.

112 Gregory C Keating, ‘Strict Liability Wrongs’ in John Oberdiek (ed), *Philosophical Foundations of the Law of Torts* (OUP 2014) 301–4 (emphasis added).

113 Goldberg and Zipursky (n 100) 765–7.

114 Arthur Ripstein, ‘Tort Law in a Liberal State’ (2007) 1 JETL, 28.

115 ibid 27.

116 Arthur Ripstein, *Equality, Responsibility and the Law* (CUP 1998) 70–2.

117 Gregory C Keating, ‘Personal Inviolability and Private Law’ (2007) 1 JETL, 6–12.

118 Hohfeld, ‘Fundamental Legal Conceptions as Applied in Judicial Reasoning’ (n 13) 760.

